# Promoting permanency in families with parental substance misuse: lessons from a process evaluation of a multi-system program

**DOI:** 10.1186/s12889-022-14528-4

**Published:** 2022-12-03

**Authors:** Karla Shockley McCarthy, Jennifer Price Wolf, Elinam Dellor

**Affiliations:** 1 College of Social Work, The Ohio State University, 1947 College Rd N, Columbus, OH 43210 USA; 2 School of Social Work, San Jose State University, One Washington Square, San Jose, CA 95112 USA

**Keywords:** Parental substance use, Family permanency, Child maltreatment and neglect, Child welfare; multi-system intervention

## Abstract

**Background:**

Families affected by substance misuse are at increased risk for child maltreatment and child welfare system involvement. The Enhancing Permanency in Children and Families (EPIC) program uses four evidence-based and informed multi-system practices to promote safety and permanency outcomes for children involved with the child welfare system due to parental substance misuse: 1) Peer Recovery Support (PRS), 2) Family Treatment Drug Court (FTDC), 3) Medications for Opioid Use Disorder (MOUD) and 4) Nurturing Parent Program (NPP) relational skill-building. The purpose of the current study was to identify barriers, facilitators, and lessons learned in the implementation of and client engagement with the main components of EPIC.

**Methods:**

Seventeen key EPIC personnel participated in the study. Individual semi-structured interviews were conducted. Qualitative analysis involved the thematic coding of the interviews, and program facilitators and barriers were revealed.

**Results:**

PRS were identified as a primary strength of the EPIC program, providing experiential connection to participating families and a valuable source of information. High turnover and matching PRS to families were barriers to PRS implementation. FTDC contributed to client success as judges developed interpersonal relationships with the clients that balanced support with accountability. Client attitudes toward court presented barriers to FTDC engagement. MOUD provided stabilization and was perceived by caseworkers as an engagement facilitator and a layer of client accountability; however, the lack of availability of MOUD service providers presented a barrier for some clients. Parental relational skill-building was not valued by clients and was perceived as conflicting with sobriety-focused activities.

**Conclusions:**

The EPIC program provides comprehensive, coordinated multi-system support and care to families affected by parental substance misuse. Continued efforts to improve recruitment and retention of PRS, reframing client perceptions of FTDC, and increasing access to MOUD may contribute to increased engagement in the program. Findings highlight the utility of tracking process outcomes in community-based interventions to promote participant engagement in programs set in complex systems.

**Trial registration:**

NCT04700696. Registered January 7, 2021-retrospectively registered.

**Supplementary Information:**

The online version contains supplementary material available at 10.1186/s12889-022-14528-4.

## Introduction

An estimated one in five children live in a home with an adult who misuses drugs and alcohol [1]. Due in part to a higher likelihood of inadequate shelter [2], lack of supervision, and impaired parenting behaviors [3], parents who misuse substances have higher child maltreatment potential than those without a diagnosed substance use disorder [4, 5]. In fact, children in these families have twice the likelihood of being at risk for child maltreatment [6]. Within the child welfare system, parental substance use is increasingly implicated in substantiated allegations of abuse and neglect [7]. Nationally, increases in overdose deaths and drug-related hospitalizations directly result in increased foster care entry [8], and substance use factored in 9.5% of all abuse cases and 12.5% of all neglect cases [9]. Child welfare caseworkers estimate that 40–80% of parents on their caseloads have problems with substance use and affected children are more likely to enter foster care [10–12].

These trends are especially relevant in Ohio, with one of the country’s highest rates of heroin and synthetic opioid-related deaths [13]. In 2017, the opioid-related death rate of 46.3 per 100,000 people was more than double the national rate of 13.3 [13]. According to the Public Children Services Association of Ohio [14], the number of children placed in custody increased by 13.5% between 2016 and 2018. In 2018, parental substance use accounted for 50% of children in state custody, with nearly half (28%) due to opioid use [14].

It is essential that families impacted by parental substance use have access to evidence-based treatment and supports to promote family unification. Although the need for evidence-based treatment is evident, barriers to care have resulted in unmet needs [15–17]. One of the most prevalent barriers is the lack of access to substance use treatment programs [15–19], and transportation was of particular concern [16, 17, 19]. The emotional turmoil from the shame and stigma associated with substance use also contributes to affected parents not seeking or receiving treatment [16–19]. In addition, financial barriers, including lacking insurance or insurance coverage, kept some from treatment [16, 17]. Not being ready to stop substance use has also been identified as a barrier to treatment [16, 17]. Furthermore, the fear of legal consequences prevented some from seeking and engaging in treatment [19].

**Fig. 1:**
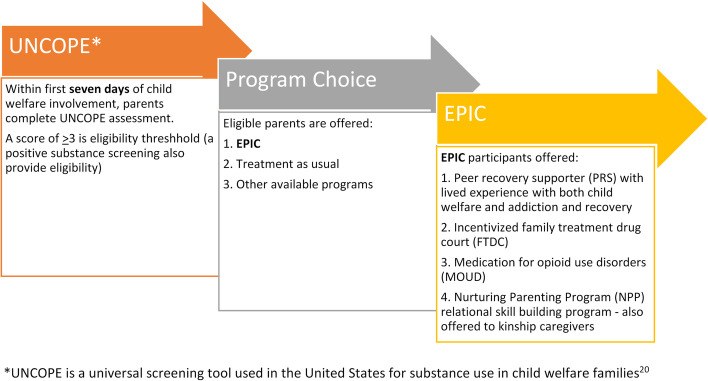
EPIC Enrollment Steps [20]

The Enhancing Permanency in Children and Families (EPIC) program is a collaboration between the [details omitted for double-anonymized peer review] College of Social Work, two child welfare agencies in central Ohio, two juvenile courts, and local behavioral health services (BHS) providers specializing in addiction treatment and recovery. EPIC’s cross-system collaboration seeks to support parents with substance misuse by reducing barriers to treatment and engagement by implementing four evidence-based and informed practices to reduce abusive and neglectful parenting, reduce parental addiction severity, and improve safety and permanency outcomes for families involved with the child welfare system due to substance misuse [5]. To coordinate services, each family is assigned a caseworker who assists the parent(s) with selecting services. The program has served 102 parents and 151 children through 4 years of implementation. The overall case flow into EPIC is provided in Fig. 1 .

### EPIC program interventions

The EPIC program offers interventions to address parental addiction and problematic parenting, with alternative judicial support and peer mentorship. Each intervention has evidentiary support, and EPIC allows families involved in the child welfare system to choose which interventions to engage, with their child welfare caseworker coordinating services [20] at no cost to the participant. The evidenced-based and informed treatments (EBTs) are 1) Medication for Opioid Use Disorder (MOUD); 2) Nurturing Parenting Program (NPP); 3) Family Treatment Drug Court (FTDC); 4) Peer Recovery Support (PRS). 

### Medication for opioid use disorder

MOUD has been found to improve treatment retention and at least double opioid-abstinence outcomes in randomized controlled trials compared to placebo or no medication [21]. MOUD reduces the physiological symptoms of opioid withdrawal without providing the euphoric state from using opioids [22]. The use of MOUD was also associated with reduced justice system involvement and increased life expectancy [23]. Despite evidence supporting the effectiveness of MOUD in promoting treatment and sobriety, the lack of available MOUD treatment programs [22, 24] significantly limits access and the number which can be treated. Additionally, the stigma attached to SUD and the use of medication to treat addiction creates a barrier to treatment that can be amplified in rural areas [24].

### Nurturing parenting program

The NPP was designed for child welfare-involved families to teach parents positive interaction skills, emotional communication, and consistent discipline with their children with the opportunity to engage their new skills with children during training [25]. Multiple studies have found NPP effective for improving parental attitudes and knowledge and reducing child maltreatment [26]. However, the lack of randomized control trials and research of the model for special populations means that the NPP is not considered an evidence-based practice and is rated a promising practice [27]. The most recently published NPP study continues to support the effectiveness of NPP in providing parenting changes that promote improved parenting, and parents also had decreases in child welfare system contact and were satisfied with their NPP experience [28].

### Family treatment drug court

FTDC involved a collaborative approach with a multidisciplinary team of judges, attorneys, child protective services, and mental health and addiction treatment professionals coordinating services to promote parental sobriety and child safety [29]. Analysis of multiple studies of FTDC found that families participating in FTDC were more likely to achieve reunification and not have an increased risk for future child welfare involvement [30]. For example, a recent study of FTDC in a rural setting found that the likelihood of family reunification and permanency were 170 and 58%, respectively, than a comparison group that did not participate in FTDC [31]. The success of FTDC has spurred growth such that there are approximately 500 FTDCs in 48 states, Guam, and the District of Columbia [32].

### Peer recovery support

PRS provided the assistance of someone with lived experience with substance use disorder who has achieved long-term recovery to establish a caring relationship with peers to connect peers to resources and provide support, motivation, and hope [33]. The use of PRS has been shown to positively impact engagement in MOUD treatment [34], faster initiation of treatment, and increased engagement and longevity of MOUD treatment for parents involved in the child welfare system [35]. Research has also found improved engagement in drug court and reduced rearrests for FTDC clients who also have PRS. In addition to maintaining long-term recovery, peers complete certification training to provide PRS; the United States has over 30,000 peer recovery supporters [36]. Peers in long-term recovery experienced with SUD, FTDC, and child welfare provide PRS for the EPIC program.

EPIC’s interventions align with the desires of policymakers to increasingly apply evidence-based treatments (EBTs) in child welfare systems to increase the quality of care and treatment effectiveness [37]. While intervention evaluations are critical for determining effectiveness, it is equally important to understand the context of interventions. Evaluating ongoing implementation efforts has the added benefit of identifying and addressing barriers to improving the delivery of programs to fidelity. The purpose of the current study was to identify barriers and facilitators to the implementation of and client engagement with the main components of the EPIC. Specifically, we assess stakeholder perceptions related to each component as follows: 1) recruitment, matching, and retention of peer recovery supporters; 2) FTDC participation and support of clients; 3) access to and engagement with MOUDs; 4) utilization of the NPP; 5) stakeholder perceived client engagement with the EPIC interventions. The interviews could provide insight into barriers, facilitators, and perceived levels of engagement to inform ongoing program implementation and future attempts to implement EBTs in child welfare contexts.

## Methods

The research followed the consolidated criteria for reporting qualitative research (COREQ) [38]. Additional file 1: Appendix A provides the completed COREQ checklist.

### Study participants

The EPIC intervention was offered in Fairfield and Pickaway counties, two neighboring counties adjacent to a major population area in Ohio. The counties share the characteristics of being similar in geographic size, household income [39], and predominately farming communities (rural), with each having one census area with over 10,000 people [40]. Both counties also have similar poverty levels, with poverty percentages of 9.4 for Fairfield and 11.7 for Pickaway [41]. However, Fairfield County has a population of over 150,000, with 87.4% White compared to almost 60,000 and 93.7%, respectively, for Pickaway County [39].

A convenience sample of 22 key personnel was invited by email to participate in the study and represented four of the five stakeholder populations: 1) child welfare administration; 2) child welfare caseworkers; 3) PRS; 4) BHS clinicians. All personnel involved in EPIC in both counties were invited to participate, and 17 agreed to participate (Table 1). Of the five that declined participation, one had left their agency, three were transitioning out of their agency, one was on leave before and during this study, and all were from Fairfield County; however, data for the positions of these five were captured by the participation of the replacement and individuals holding the same positions. Participants were White (100%) and predominately female (94%). As shown in Table 1, each position has at least two participants and represents the majority of personnel involved in EPIC. Unfortunately, the fifth stakeholder population of FTDC personnel was unavailable during interviews to participate in this study.

**Table 1:** EPIC process evaluation participants

Child Welfare Partners	Fairfield County	Pickaway County	Total	Percent of Total EPIC Personnel per Position
Child Welfare Administration	1	2	3	60%
Child Welfare Supervisors	2	1	3	100%
EPIC Caseworkers (Child Welfare)	4	1	5	83%
Peer Recovery Supporters	3	1	4	80%
**BHS Partners**^a^ BHS Supervisors	**Ohio Guidestone**	**Integrated Services**		
	1	1	2	67%
**Total**	11	6	17	77%

^a^Behavioral Health providers specialize in addiction treatment and recovery and serve both counties

### Data collection procedures

Headed by the lead author, a team of three social work students conducted in-person, one-on-one semi-structured interviews beginning late October 2019 through early February 2020. All interviews took place in the offices of child welfare or BHS agencies. Participants provided written informed consent and agreed to the audio recording of the interview; the interviews lasted approximately 1 h. The EPIC project director developed the interview guide to reflect the efforts of each stakeholder as described in the EPIC protocol, case flow of program enrollment and engagement, and memoranda of understanding. Questions were structured to elicit information concerning barriers and facilitators to component implementation and client access and engagement, as well as overall perceptions of each component. The guide was reviewed for appropriateness and completeness by the entire research team involved in EPIC project efforts and, thereby, had detailed information about stakeholder EPIC actions and responsibilities. Interviews began with questions related to recruitment, enrollment, and engagement in the program as a whole. All stakeholders were asked questions about EPIC training (readiness) and implementation. Stakeholders were also asked a series of open-ended questions specific to their role in EPIC and serving their population. Child welfare administration and child welfare workers and supervisors were asked questions about implementing the four interventions and the barriers and facilitators to client engagement. Service providers administrators were asked about the Nurturing Parent Program. Further, PRS were asked about their client interactions and detailed questions about client engagement with FTDC and MOUD. Caseworkers and PRS were also asked about collaborating with clients and with each other. Additional file 2: Appendix B presents the complete interview guide.

### Data analysis

Interviews transcriptions and field notes were reviewed and independently thematically analyzed by two authors using ATLAS.ti [42]. The authors identified significant phrases from each transcript about participants’ experiences to formulate meanings. These meanings (codes) were clustered into themes allowing for the emergence of patterns common to all participants’ transcripts [43]. After coding all data, the authors created a table via Microsoft Word to facilitate data interpretation. Relevant quotes were selected, and naturalistic generalizations were summarized during team meetings among all authors.

The sample size of 17 interviews falls within the findings of the Hennink and Kaiser [44] of needing 9–17 interviews to reach saturation. In addition, the approach recommended by Guest et al. [45] was followed to determine the adequacy of the sample size after data collection. The Guest et al. method involves first setting a new information threshold and establishing a base size to represent the minimum number of interviews to provide the base of information already gained on which to compare. For this study, the new information threshold of zero was selected and the base size set at five interviews – one from each stakeholder category. The next step is to determine the run length or the number of interviews to compare to the base for new information. The run length was set at five, and interviews were selected from the other county for each stakeholder category that was not included in the base. Qualitative analysis of the base resulted in the emergence of 17 codes (Step 1). The 17 codes encompass all four interventions as well as the EPIC program implementation. The run did not result in additional codes, but did result in the final code inclusion for each stakeholder group. According to the Guest et al. method, data saturation was reached with the base interviews, making the sample size of 17 interviews more than adequate. Table 2 shows the saturation assessment.

**Table 2:** Saturation assessment

***Interview number***	***1***	***2***	***3***	***4***	***5***	***6***	***7***	***8***	***9***	***10***	***11***	***12***	***13***	***14***	***15***	***16***	***17***	***Total***
New codes (base)	10	1	4	2	0	0	0	0	0	0								17
New codes (run)	17					0												0
% change over base										0%								17

## Results

The results of our thematic analysis are summarized in Table 3, with Table 4 providing a detailed summary of themes by intervention. The findings, supported by participant quotes, are provided below for each program component – PRS, FTDC, MOUD, and NPP. For ease of reading, fillers and false starts (e.g., um, you know) were removed from quotes except where needed to maintain meaning.

**Table 3:** Summary of themes

Implementation Barriers	Implementation Faciliators	Client Engagement Barriers	Client Engagement Facilitators
• Relationships and Previous Experiences • Control of PRS Employment • Lack of MOUD Service Providers	• Relationships • Structure and Stability	• Transportation • Mandatory Child Welfare Involvement • Relationships and Previous Experiences • Lack of MOUD Service Providers • Parenting Intervention Unnecessary	• Relationships • Structure and Stability • Incentives

**Table 4:** Summary of themes and details by intervention

Peer Recovery Support (PRS)	Family Treatment Drug Court (FTDC)	Medications for Opioid Use Disorders (MOUD)	Nurturing Parent Program (NPP)
• Facilitators ° Impactful relationships with clients ° Connect caseworkers to clients ° Provides purpose to PRSs • Barriers ° Maintaining boundaries ° PRS turnover ° Matching PRS and client ° Not employed by child welfare	• Facilitators ° Provides structure ° Provides accountability & support ° Relationship with judge ° Incentives • Barrier ° Court viewed as punitive	• Facilitators ° Use shows motivation ° Stabilization • Barriers ° Knowledge of MOUD ° Availability of providers ° Preference for Subutex	• Barriers ° Sobriety efforts take precedent ° Not valued ° Redundant with FTDC
**Other Barriers to Engagement**	• Transportation • Client resistance ° EPIC program not voluntary ° Client not ready for change ° Client animosity toward child protective services		

### Barriers

The participants provided client barriers that were universal to the EPIC program as well as specific to the individual interventions within the program. In addition, barriers related to the implementation of the programs were also discussed. Both client barriers (e.g., transportation) and implementation barriers (e.g., lack of service providers) ultimately affected client engagement.

#### Transportation

The most discussed universal barrier to treatment for both counties was the lack of transportation. Transportation impacted clients’ ability to get to FTDC and keep meetings with protective services and service providers, thus affecting every EPIC intervention except PRS. Although PRS provided transportation to clients for EPIC-related services, caseworkers reported that lack of planning added to transportation problems:

Transportation is a bit of a barrier here. Just because…our public transportation system is not reliable. So, if you live in Circleville, a lot of the clients could walk, they just don’t want to. So transportation gets to be an issue, but the people that we work with, our peer mentors are great at getting our clients to where they need to be. It’s just them using the resources again. If you reached out to the peer mentor, we’d get your ride. It’s just they wait to last minute to call.

Another caseworker added:

A lot of it is transportation, or just not remembering their appointment. We’ve given ‘em planners. We’ve given ‘em reminders. So a lot of it is just not them utilizing their resources to get a ride…not telling me when the appointment is so I can schedule one for them. That’s really the only barrier, because they’re really good at rescheduling for ‘em. If they do miss it, and then it’s again, just not telling me when the appointment is.

#### Mandatory child welfare involvement

Being involved in the child welfare system was not a choice parents make; it arose when the well-being of children was at significant risk. If parents wanted to keep or regain custody of their children, they had to navigate the child welfare system and meet change milestones. Caseworkers explained how this forced involvement with child welfare caused client resistance to treatment and a lack of client buy-in. One caseworker described this resistance and the need for additional efforts to promote client acceptance:

They keep on saying, well, EPIC isn’t voluntary. And I’m like, yes, to a certain extent, compliance is a big thing. So you want to ask them if they’re going to actually stick with it, so I’m not hunting down people week after week after week who are hiding from me, who really don’t want to be in the program, but you just said you’re gonna be in it… A lot of them are grateful and didn’t realize that there were these services provided for them. Everybody has a stigma. Children Services coming in, taking your child. So letting them know we’re here to work on this one on one, really working on what your needs are.

This caseworker further explained that lack of client engagement in child welfare services occurred when clients did not acknowledge problems and, therefore, were not ready to begin working toward sobriety, saying, “I think a lot of it’s just the readiness piece, just denying that there’s any substance abuse disorders or any substance abuse problems.”

#### Relationships and previous experiences

It was not unusual for clients to have had previous experience with the child welfare and court systems. These previous experiences resulted in some clients having adversarial relationships with children protective services caseworkers that presented barriers to client engagement in EPIC. In addition, prior court experience left many clients regarding the courts as punitive, causing them not to be interested in FTDC.

Resistance was noted when clients did not consider the caseworker and EPIC interventions as allies. As explained by a caseworker, it was difficult to engage clients when in addition to not being ready for change, they view EPIC interventions from a combative, instead of supportive, stance:

Some people just aren’t ready to be sober, and that’s just one of the barriers. It’s nothing that any program can really help with. Sometimes I think it just primarily is they don’t want to be sober…The ones that I have in the program, and…have been engaged, stay engaged, because they really want it and they understand all the resources.

A PRS also found that some clients viewed protective services as the “enemy.” As a result, this PRS emphasized the need to educate clients about the purpose of child protective services and EPIC:

And even with CPS (child protective services), because everyone has this [idea] that they’re the enemy. They wanna take my kid. That’s the last thing they wanna do... it’s a lotta paperwork and a lot of—They don’t wanna do that, you know. And, so once I came into this and really saw, we have a really good protective services. They’re so awesome, and people end up seeing that. They really do, but they have had a bad experience in the past, because it used to be that way, so I think that people’s views are really changing.

Previous experience with the court system made some clients uneasy about participating in FTDC and often required persuasion by caseworkers and PRS. A PRS discussed the additional effort needed to help clients reframe their understanding of court systems:

A lotta times the participants are just hesitant about the word “court,” period. They’ve not had the best experience in court always…but one of the selling points I would always make…is that this is an opportunity to go in front of the magistrate and really show progress, like, every week, and then it goes to two, then three, rather than only once every 3 months. And I always sold it as a real opportunity to establish that professional relationship with the magistrate and the court people.

Relationships also affected PRS-client assignment. Caseworkers explained that screening and matching participants with the appropriate PRS could be challenging and impact client engagement, especially in a small town. For example, one caseworker described these challenges due to the popularity of a PRS peer:

Shannon* knows everyone, so that can be a problem sometimes. And it depends on how she knows them. As soon as the name’s mentioned, she’s like, oh, I know them. Well, how do you know ‘em? We have to go through that whole—Like, it’s every time. She is also, I think just a few years into sobriety, where I’m 10, so I’m very far removed from that world. (*name changed)

Furthermore, participants sometimes refused to be matched with peers with whom they did not identify. For example, one caseworker reported one participant saying, “I’m not working with a male.” A second caseworker related that when a peer recovery supporter declined to be matched with a client, “She’s like, I don’t have anything in common with this guy*.”*

The well-being of PRS, ability to support clients, and job retention somewhat depended on their interactions with clients. The ability of the PRS to develop supportive relationships with clients and use their experiences navigating the child welfare and court systems to help clients do the same defined the PRS role. However, establishing and maintaining healthy emotional boundaries was challenging, and their clients’ struggle with sobriety and desire to be with their children can transfer to the PRS. When PRS could not maintain those boundaries, their own sobriety was at risk. A PRS describes one such situation:

So she has her kids back. It’s crazy though, so I need to talk to someone today about it because her kids are - ooh, it’s rough. I don’t know… I’m worried for her because I think I would use in that - I mean, it’s, − that’s weighing heavy on me today. Oh, I take it home. I really do, and I try so hard not to, but man, it’s hard.

A final barrier that arose from previous experience was clients’ belief that the process of accessing services was complex and the services themselves were complicated. A caseworker explains this phenomenon regarding MOUD and the need to counter this belief to encourage client engagement in treatment:

You don’t really have to do anything more. Like, I don’t know if they—there’s a stigma or this thought that they have to do all this— jump through all these hoops to get their medication. And I’m like, no, you don’t have to really do anything more than what you’re already doing.

#### Control of PRS employment

County caseworkers expressed that having PRS employed and managed by BHS agencies created barriers to implementation and increased disruptive PRS turnover. A caseworker relayed how having BHS agencies, instead of child welfare agencies, employment of PRS created instability, discontinuity of service, and added stress:

I’ve had a lot of issues with this. When I started we had two peer mentors who, I guess EPIC gave the money to [BHS agency], and so the two peer mentors were through [BHS agency]. Everything was going really well. Loved our peer mentors. Well then, they kind of went and just told one of the guys you’re part-time the day before. So, then it kinda—we were down to one and a half peer mentors that me and the [another program] worker were sharing. So then they decided to divvy them up. The one would go with [another program], one would go with EPIC. That was working until the one peer mentor was like, no, I’m not gonna do this anymore. So then we were down to one peer mentor, sharing EPIC and [another program].

Another caseworker reiterated the challenges to case management and client stress related to turnover rates and matching PRS to clients’ needs.

It’s been difficult with the peer mentors. And I know the peer mentors have had some stress about it. Like they don’t understand their job title, because they keep switching around so much. So I don’t know... And I know my clients are better with it, ‘cause now they’ve gone through three different peer mentors. They’re like, “So who am I supposed to be calling?”

Relationships of caseworkers with PRS and PRS with clients were tenuous and affected providing continuity of care. County caseworkers were frustrated by the investment of time in training and building relationships with PRS when disrupted by staff turnover and novice replacements. These changes also interfered with client-PRS relationships and caused client setbacks, feelings of abandonment, and treatment disengagement. One caseworker stated:

So now I’m just paired with this new peer mentor who has never done this before. She just started probably 3 weeks ago. So now I’m on top of trying to take care of all my cases, I’m trying to train her. And she just follows me around everywhere I go, so it’s kinda stressful. We had such a good role going with the other ones… So we were on a great roll and then we’ve had this disruption, where we’re kinda trying to get back into the swing of things. I’m, still confused on her purpose… It’s been difficult with the peer mentors. And I know the peer mentors have had some stress about it.

#### Lack of MOUD service providers

Caseworkers and PRS commented on how the lack of MOUD service providers interfered with client engagement in the MOUD intervention and with the supervision of services by caseworkers when clients find their own MOUD providers. One caseworker discussed the challenge of case management when clients opt for MOUD through service providers not connected to their agency:

I know some of my clients go to some strange places for MOUD, where I don’t [know] where you found these people? But it’s hard to communicate with those people as well, because some of the treatment providers are like, nope, they didn’t sign a release here or they won’t call you back. So it’s just difficult all around with every treatment provider.

The lack of available MOUD services made clients’ progress toward sobriety more difficult. A PRS stressed the importance of accessing MOUD within the clients’ window of readiness to support their addiction recovery:

I would have to say one of the barriers is getting people in fast enough. Because if you don’t have someone stabilized, they’re not gonna, I mean, we’re pretty good about it. Not [BHS agency]…I hate to say it, but they kind of—they don’t run their end because they’re not getting people in fast enough at all, and if they do, it’s in Chillicothe or Columbus, which, I’m willing to do that, take them, get ‘em in, but the caseworkers just stop referring people there because they couldn’t meet the need. So I think it’s gotten a little bit better. We have another prescriber now. I think that’s gotten better, but you gotta be fast with that. I mean, that’s gotta be quick if you want someone to really quit.

Another issue challenging client engagement is the lack of MOUD providers that dispense Subutex.

A PRS explained that clients often prefer Subutex to Suboxone as it does not contain naloxone and therefore does not block the opioid euphoric effect. Furthermore, this peer recovery supporter warned that Subutex has a street value that may encourage misuse:

One of the issues I see with a lotta people are they want Subutex…Suboxone has naloxone in it, and that is the blocking agent, and then just straight buprenorphine is, like, the craving part of it. That’s a problem…They only come in pills, not films, so high abuse rate - selling. I think that they go for more on the street, is what I hear, so that’s a problem...But I have a lotta people on it, and…they don’t need to be on it… people go to cash doctors, because they’ll give them the Subutex when they have Medicaid, and I wanna pull my hair out. I’m like, no. And it’s an hour away. No, right here, Medicaid, go. Yeah, that’s part of their addiction. It’s being in control, and they’re not willing to let go of.

A county caseworker detailed the challenge of clients shopping MOUD providers looking for Subutex:

I think the challenge that we have is that there’s some people that go outside of those service providers, that the cash-only type things, and we really encourage people, − I don’t know if it’s a control thing for some people. They’re like, this is how I’ve always done it. You can’t tell me what to do kinda thing… And I think people like these cash doctors will give them the Subutex as opposed to the Suboxone. But now I’ve had a couple of people that their cash doctors are not giving the Subutex anymore.

#### Parenting intervention unnecessary

Both counties reported that their clients had little interest in the NPP relational skill building intervention. Instead, clients prioritized sobriety-related activities and considered the NPP to be an unnecessary burden that detracted from attending to working toward sobriety. Clients also thought the NPP to be redundant; a caseworker explained that the FTDC program already incorporated a parenting class as part of the process, so a separate intervention was not necessary:

I don’t have a lotta people in it …The only thing I can say about that is I know, in FTDC, once you’re in step three, you have to have some type of parenting class, so I feel a lotta people will do that. Once my people move up further because it’s the easiest one to do because it’s already linked with someone that they know.

Another caseworker provided a different issue contributing to parents not engaging with the NPP program - some parents felt they already knew how to parent their children properly:

I think that they feel like they know how to raise children, that they don’t really need that extra support, that extra help. I always just approach it as, well, this is just trying to help you, educate you more about the drug disease and the addiction and how you can improve your relationship with your adult child. I haven’t had much success. People just don’t wanna do it.

### Facilitators

Participants detailed that supportive interpersonal relationships was the driving force of client engagement. The structure and stabilization provided by the EPIC interventions also contributed to implementation and client treatment involvement. Some participants also found that clients were motivated by incentives offered by the EPIC program.

#### Relationships

Although existing relationships clients had with child protective services and courts presented barriers to client engagement, the positive, supportive relationships fostered among the EPIC stakeholders and clients during the EPIC intervention proved the greatest strength in promoting client engagement. Clients had not had a PRS before, which made it easier to develop those relationships. However, more effort was needed to encourage clients to engage with FTDC and the support provided by the judges.

Caseworkers universally viewed PRS as a strength of the EPIC program. For example, a child welfare administrator stated, “I think the peer recovery coaches have made quite an impact.” PRS have the lived experience of child welfare and court involvement that allowed them to connect with families at a deeper level. The administrator further explained:

When we first started all this… I didn’t know how successful it would be. You hear that it’s worked in other states, but, you know, is it really gonna work here? And it’s been a great process. Our caseworkers can say the exact same thing that the peer recovery coach says word for word, but there’s credibility in the peer recovery coach saying it because they’ve been down that path. I think it’s very good.

Caseworkers viewed PRS as a valuable resource that bridged the gap between child protective services and the reality of being a parent with an addiction trying to unify their family. One said, “I feel that we work well with our peer mentors, that we rely on them a lot to make those connections with the families.” Others echoed this sentiment, explaining that PRS’ struggles with addiction and child welfare involvement give them the legitimacy and ability to help families navigate both systems while also serving as a source of support in coping with the emotional turmoil brought on by these circumstances.

Recognized as the most critical component of the EPIC program, PRS well-being, and job commitment are crucial. Although client engagement could be detrimental for some PRS, others reported that working with EPIC participants was beneficial. They felt a sense of purpose, knowing they could leverage their past experiences to help others. One PRS discussed how the role of the PRS in EPIC removed the stigma of addiction for the PRS and showed clients the other side of addiction:

I think the whole program is great. I think this is an amazing thing. Um, I think that, for too long, the one—the addicts, the ones of us that would get passed addiction and would go on and lead fulfilling lives, we always had this secret in our back pocket that we didn’t wanna anybody to know about, and that has—it’s kind of gotten rid of that. It’s okay to say, “Yes, this is part of my life, and I—and this happens, and I did this. But, look, I’m okay.” And-and I—and by being able to be okay and be open about it, we’re making it available to so many more people, and so I think it’s an amazing thing. It’s an amazing thing. I really do.

Another PRS explained that her negative perception of county child protective services changed throughout her work and made her better positioned to help clients:

Once I came into this and really saw we have a really good protective services. They’re so awesome, and people end up seeing that. They really do, but they have had a bad experience in the past because it used to be that way, so I think that peoples’ views are really changing. When we got the case, they said, “oh, they’ll never work services. We’re just gonna take permanency.” And they have a brand new baby, seventh baby, and they got their other baby back in the home, and they see their other kids. So it’s just—yeah. Like, it melts my heart. It really does. They’re getting ready to close, too. The case is about to close. …

Additionally, a third PRS found the benefits of building supportive relationships with clients and sharing experiences by being a PRS so personally helpful that she encouraged a client who successfully completed the EPIC program to become a PRS:

I do have a client that finished the program, and I got her a job. She’s gonna be a peer mentor. I know, right? That’s pretty cool. …I thought she’d be a great peer mentor. She brings a great story. A different, good complement to what is already here.

The interpersonal relationships clients built with FTDC judges also motivated client engagement. Caseworkers noted that EPIC and FTDC provided an opportunity for clients to develop positive, supportive relationships with authority figures clients may not have otherwise had:

I think the benefits are keeping them engaged. Going in front of the judge weekly, we stress it. Like this is your time for the judge to get to know you, and your strengths, and what you’re doing well. They really enjoy that, because they do get a different bond with a judge that they wouldn’t have in normal court…and he says it to ‘em too, like this is your time to voice your concerns when you’re afraid to with all your attorneys here… they get time with their kids more often, and get to increase that, and they see reunification happening way sooner. And I think that keeps them very motivated in this whole process.

#### Structure and stability

Coordinating multiple interventions through child protective services to support parents through family unification and sobriety was considered advantageous for clients. The EPIC program structure allowed caseworkers to more effectively connect clients to mental health, medical, and legal supports that all worked together. The EPIC program’s approach to care promoted client engagement by being responsive to clients’ needs. One PRS remarked about the efforts put forth by one county to engage parents and encourage sobriety and family unification:

XX County is very big on whatever it takes. Um, CPS, the courts, they’re all—I mean, everyone’s on the same page. They don’t really care what it is, whatever we have to do to get you there, um, which is a beautiful thing,

The structured framework provided by FTDC was praised for giving clear metrics for completing case plans and eventual reunification with children while reducing the barrier of the legal consequences of parental substance use. For example, one caseworker described the court process and how the expectations of the judge encouraged clients to take responsibility for their experiences and progress:

… we make our referral to the court coordinator… then she (court coordinator) will do the initial interview with them to make sure that they are a fit for the program. Once she decides that they’re a fit for the program, they have to sign off with their attorneys, and then they’ll start.

They have a strict program they have to follow. They go to three A.A. or N.A. meetings in the first phase a week. They have to meet with me. They have to meet with their counselor and peer mentor, as well as random drug screens… And they do two of those a week…and a lot of the times if they’ve missed a phone call or miss a drug screen, they write a paper on accountability, and we’ve even sanctioned all the way up to jail time for noncompliance with the program… Once they’re in it, though, they seem to thrive and understand the accountability part and enjoy the rewards from it.

Some of the barriers are just clients are scared of the sanctions, which I completely understand. At the beginning, if they’re not completely engaged, they’re hesitant. I have noticed after the case is open for a while is when they start thinking, this is probably the better option for me, because they haven’t done anything in the first couple months. And then the judge is kind of on ‘em, like you’re coming to the end of the time we allow for you. So the only real barriers is just themselves not being ready or the sanctions that comes with it.

Another caseworker discussed how FTDC provided a stabilizing, supportive experience for clients:

I think that our family court is very helpful, that it wraps those supports around everybody,—our family court has a case manager that works directly with the family, gives us updates. I think the families that report back to me that like family court, that it’s all very positive. It’s not like a punishment or sanction driven.

Another intervention, MOUD, provided structure through medication control and testing. Because of this increased oversight, caseworkers considered opting into MOUD a positive indicator of clients’ level of motivation and engagement to achieve sobriety.

We leave it up to them for MOUD….I mean I don’t stress it with ‘em. I do make sure that if they enroll in MOUD that we are keeping track of their prescription. But really, I mean if they’re in the program, they’re pretty consistent with going to those appointments, and because they know they’re gonna be screened twice a week [laughter] and they should have that in their system. So, we don’t have to encourage them too much about being consistent.

A second caseworker explained that stabilization while receiving MOUD was the primary motivating factor for client engagement.

I feel like engagement isn’t an issue in that section because getting their MOUD services is what’s helping them stay clean for the most part, and most people if they want a referral are gonna want to do it. So, I feel like it’s different than like, “Hey, go do a drug screen today,” because that’s not something that most people wanna do, but the MOUD services are I would say.

#### Incentives

FTDC encouraged client engagement by incentivizing clients to participate and meet goals. A PRS describes how the court set weekly goals, and clients received various incentives for meeting goals and a continuation of goals not met:

They have all these incentives that they do for—I mean, every week, each, like—so I have three people that are in it now. Every week, they’ll have a goal that they have to meet, and if they don’t meet it, then we’ll try again next week. If you do it—if you do meet it, then they get an incentive, whether it’s a gift card or, like I said, family pictures being done, or there’s board games for kids, I mean, like, all different things.

In addition to various items, financial incentives were a part of FTDC. One caseworker spoke to the motivation provided by the financial incentive to participate in drug court under EPIC:

…we’ve had a lotta people that have had a bad experience. Because before, the judge would sanction, send ‘em to jail. Now, they don’t do that. For me, an outsider looking in, I always tell people, it’s more support. And I hate to say, like, financially, but there is financial help there. Like, take it. You need it. You’re checking in once a week for a couple weeks. Then once every 2 weeks, and you have this support, and they are really supportive. Like, I get a little jealous sometimes. I’m like, man, can you pay a bill for me, you know. It’s super supportive.

## Discussion

In the present study, we aimed to identify barriers and facilitators to the implementation of and client engagement with the main components of EPIC and to identify lessons learned to improve the delivery of the EPIC program. Specifically, we assess stakeholder perceptions related to each component as follows: 1) recruitment, matching, and retention of peer recovery supporters; 2) FTDC participation and support of clients; 3) access to and engagement with MOUDs; 4) utilization of the NPP; 5) stakeholder perceived client engagement with the EPIC interventions. We interviewed 17 stakeholders - child welfare and BHS providers, front-line caseworkers, and PRS working directly with EPIC participants – from two counties in a Midwestern state. Participants detailed barriers and facilitators to engagement in treatment.

Participants agreed that transportation presented a significant barrier to client engagement. This challenge, which aligned with findings from other studies [16, 17, 19], caused clients to miss FTDC and MOUD appointments and meetings with caseworkers and PRS. Missing appointments could delay treatment and have consequences in court. PRS were instrumental in helping participants overcome transportation challenges to meet EPIC program obligations and support basic needs such as grocery shopping. However, clients not providing timely transportation requests or remembering appointments continued to be a problem. PRS related that improving communication among caseworkers, PRS, and clients about scheduling in advance would improve their ability to support transportation needs of participants.

Similar to other studies [16, 17], client resistance impeded client engagement and service delivery. Involuntary child welfare involvement fostered client resistance, particularly for clients who did not acknowledge they had a problem and were not ready to address substance use. Prior adversarial and punitive experiences with child protective services and the court also contributed to client resistance to engagement in EPIC interventions. PRS were instrumental in educating clients on the intent of the EPIC interventions, especially the difference between FTDC and other courts clients have experienced, to help clients realize the value of engaging in services. Caseworkers and administrators further explained that because PRS have shared experience navigating addiction treatment and child welfare systems, they communicate information and engage participants in a way that caseworkers are unable.

In addition to navigating the multiple systems parents with substance use encounter, EPIC PRS helped participants work through the emotional turmoil from the stigma and shame associated with parental substance use [16–19]. This revelation aligns with previous research showing that PRS are likely to relate better to challenges that parents face and, as such, offer them a hopeful outlook [46]. However, the critical support provided to clients through positive relationships with PRS was at risk as implementation barriers limited PRS service delivery quality. There was a disconnection between BHSs’ management of the PRS workforce and supporting clients. Caseworkers were particularly vocal about PSR employment management contributing to PSR turnover and disrupting the essential relationships between PSRs and clients. Caseworkers argued they would have improved working relationships with PRS, there would be lower PRS turnover, and clients would enjoy better continuity of care if child protective services managed PRS employment. Furthermore, caseworkers believed they would employ and retain more PRS resulting in improved matching of PSRs to clients.

Respondents were also overwhelmingly positive about FTDC and the additional accountability and support provided for clients. Successful FTDC participation involved helping clients overcome their fear of legal consequences [19], primarily from prior, mostly punitive, experiences with court systems. This process and the resulting ability of the participant to form trusting relationships with judges echoes similar findings in existing studies. For example, Lens [47] noted the importance of judges building collaborative and respectful environments and using empathy and support to create more therapeutic environments. Therapeutic jurisprudence had a positive recovery impact on participants [48]. The relationships built with judges and staff allowed participants to tell their stories, feel respected, and build trust [47, 48] in a way that supported participants as they developed long-term recovery and wellness [48]. Furthermore, the financial incentives offered for participation helped with financial issues that can be a barrier to treatment [16, 17].

The structure and stabilization provided by MOUD contributed to client engagement. MOUD promotes recovery for parents involved with child welfare [49]; however, not all EPIC participants engaged in MOUD. One issue may have been that EPIC participants were not aware of the availability of MOUD or thought participation in MOUD would be complex and cumbersome. The most concerning barrier to MOUD was the lack of available providers, especially when clients were ready to engage. The scarcity of MOUD providers was an established problem not unique to the EPIC program [15–19, 49].

EPIC offered the Nurturing Parent Program (NPP) to help parents develop parenting and relational skills. NPP can improve parenting efficacy and lower the rates of subsequent investigations for child welfare-involved parents [28]. However, NPP was the intervention parents least engaged. The complexity of dual treatment of substance use disorders and parenting was not specific to the EPIC program. The different priorities and treatment goals presented challenges that prioritized or sequenced treatment [50]. EPIC study participants related similar circumstances, indicating that the lack of NPP engagement may reflect prioritization of sobriety over other identified goals. FTDC also includes a parenting component, possibly making NPP redundant. There was also a sentiment that EPIC-involved parents perceived their substance use as the problem and did not need parent training.

### Lessons learned and future directions

Relationships were the most critical component to EPIC client engagement and increased successful client sobriety and family unification. Meaningful interpersonal connections PRS made with clients helped clients overcome multiple barriers to engagement in treatment. PRS bridged all of the interventions and connected with clients at an experiential level that no other participant could achieve. However, there were insufficient PRS to meet the demand, and turnover was problematic. Efforts are needed to increase and retain the pool of PRS, especially in smaller counties. Further study could uncover factors that promote and inhibit those in recovery from becoming PRS. This knowledge would inform recruitment strategies among participants who successfully navigate addiction treatment and child welfare systems. Increasing the pool of available PRS should result in improved PRS-client matching by providing choice among PRS. Investigation of factors contributing to PRS turnover would provide areas of change to improve the retention of PRS. This study also revealed communication challenges and incongruence between the BHS providers that employ PRS and the child welfare agencies that utilize PRS services for their clients. Either improving collaboration and developing shared goals and vision for PRS or transferring employment management to child protective services agencies may provide more stability and consistency.

Additional efforts were needed with clients with previous contact with child welfare and courts as they were less likely to engage in EPIC interventions. PRS emphasized the need to educate clients about the intentions of EPIC as coordinated support for parent sobriety and family unification to dispel beliefs that child welfare and court systems are working against parents. Intentionally assessing clients’ attitudes toward child protective services and the courts at the start of the EPIC intervention could better equip PRS, caseworkers, and judges to combat associated client adversity and possibly result in earlier and more dedicated client engagement.

MOUD was a positive stabilizing option for parents working toward sobriety, but the lack of available providers was problematic. Clients also had misconceptions about the complexity of engaging in MOUD treatment. Providing additional education about MOUD should help remove confusion about the treatment. Broadening the network of reputable MOUD providers is more complicated as EPIC provides funding for MOUD treatment, but adding MOUD treatment providers is beyond the scope of EPIC. However, continual evaluation of the county and near-county landscapes for additional reputable MOUD providers could increase the number of providers that should improve the service delivery and utilization of MOUD for treating parental SUD.

## Limitations

Several study features may limit the interpretation or generalizability of our findings. First, this study focuses on the experiences and perceptions of stakeholders implementing the EPIC program; thus, clients were not included. The lack of the client perspective to understand facilitators, engagement, and access may limit the applicability of these findings to other contexts and settings. Second, we could not interview FTDC staff directly; while caseworkers and PRS could speak to this intervention, obtaining this perspective would undoubtedly increase understating of barriers and strengths from their viewpoint. Third, the current study exclusively focused on the EPIC program components. Fourth, the stakeholders and the participating counties are predominately White and do not reflect the over-representation of Black and Indigenous families, and the increasing trend of Hispanic families, in the child welfare system [51]. Therefore, the findings may not be generalizable to other interventions, treatment systems, or locations. Furthermore, the affiliation of the interviewers with the university partner of EPIC may have impacted the participants’ willingness to discuss experiences openly. However, the interviewers were not involved in the implementation project and did not know the interviewees, reducing the risk of interviewer bias.

## Conclusion

EPIC provides a comprehensive collaboration of child welfare workers, judges, court staff, and peer mentors that shows promise to help promote parent recovery, sobriety, and family unification. EPIC centers intervention efforts on positively supporting the needs of the parent and family while the parent actively participates in treatment planning and execution. This study highlighted that stakeholders perceive the program’s strength as fostering supportive relationships among the stakeholders and with clients; further research is needed to capture the client perspective to understand better how interpersonal relationships impact client engagement and outcomes. In addition, the EPIC program will benefit from continued efforts toward communication, program education, and peer mentor recruitment and retention to improve service delivery to these families in need.

## Supplementary Information

**Additional file 1: Appendix A.** COREQ (Consolidated Criteria for Reporting Qualitative Research) Checklist [24].

**Additional file 2: Appendix B.** EPIC Process Review: Interview Questions.

## Data Availability

The dataset analyzed during the current study is not publicly available due to the protection of participant privacy but may be available from the corresponding author on reasonable request.

## Abbreviations

BHS: Behavioral Health Services
CPS: Child Protective Services
EBT: Evidenced-Based Treatments
EPIC: Enhancing Permanency in Children and Families
FTDC: Family Treatment Drug Court
MOUD: Medications for Opioid Use Disorder
NPP: Nurturing Parent Program
PRS: Peer Recovery Support
RSB: Relational Skill Building
