# Supplementary material for: Promoting permanency in families with parental substance misuse: lessons from a process evaluation of a multi-system program
Source: BMC Public Health. 2022 Dec 3;22:2261. doi: 10.1186/s12889-022-14528-4 (PMC9719642; doi:10.1186/s12889-022-14528-4)
Supplement: Supplementary file 1 — Additional file 1: Appendix A. COREQ (Consolidated Criteria for Reporting Qualitative Research) Checklist [24]. [file 12889_2022_14528_MOESM1_ESM.docx]

**Appendix A**

**COREQ (Consolidated Criteria for Reporting Qualitative Research) Checklist** (24)

| **Guide Questions** | | **Response for This Study** | **Section** |
| --- | --- | --- | --- |
| **Domain 1: Research team and reﬂexivity** | | |  |
| ***Personal characteristics*** | | |  |
| Which author/s conducted the interview or focus group? | Interviews we conducted by the lead author, two additional Ph.D. students, and one bachelor's student; the second and third authors did not conduct interviews |  |  |
| What were the researcher's credentials? | Interviewers: BSW, MSW  Authors: MSW, Ph.D. | Title page, Methods |  |
| What was their occupation at the time of the study? | Interviewers: social work Ph.D. and bachelor's students  Authors: social work Ph.D. candidate, social work Ph.D. faculty, social work Ph.D. senior researcher | Title page, Methods |  |
| Was the researcher male or female? | female |  |  |
| What experience or training did the researcher have? | Lead and third authors have conducted many focus groups and individual interviews for qualitative analysis; all Ph.D. students had coursework in qualitative research methods, and the bachelor's student was provided interview instruction by the lead author |  |  |
| ***Relationship with participants*** | | |  |
| Was a relationship established prior to study commencement? | The third author had a professional relationship with the participants and did not conduct interviews or analyze the data; the remaining authors and interviewers did not have a relationship with the participants |  |  |
| What did the participants know about the researcher? | Participants were briefed on the purpose of the study and understood that it was a research project conducted by [details omitted for double-anonymized peer review] concerning the implementation of the EPIC program | Methods |  |
| What characteristics were reported about the interviewer/facilitator? | The interviewers were all affiliated with the university partner of the EPIC program, which may influence participant responses | Limitations |  |
| **Domain 2: Study design** | | |  |
| ***Theoretical framework*** | | |  |
| What methodological orientation was stated to underpin the study? | thematic analysis based on a descriptive phenomenological | Methods |  |
| ***Participant selection*** | | |  |
| How were participants selected? | Purposeful sampling of key stakeholder personnel | Methods |  |
| How were participants approached? | Invited to participate via email | Methods |  |
| How many participants were in the study? | 17 | Methods |  |
| How many people refused to participate or dropped out? Reasons? | 5 refused/not available | Methods |  |
| ***Setting*** | | |  |
| Where was the data collected? | At child welfare and behavioral health agencies | Methods |  |
| Was anyone else present besides the participants and researchers? | No |  |  |
| What are the important characteristics of the sample? | County location and occupation | Table 1 |  |
| ***Data collection*** | | |  |
| Were questions, prompts, guides provided by the authors? Was it pilot-tested? | Interviews were semi-structured; guides are provided in Appendix B | Appendix B |  |
| Were repeat interviews carried out? If yes, how many? | No |  |  |
| Did the research use audio or visual recording to collect the data? | Audio recording | Methods |  |
| Were ﬁeld notes made during and/or after the interview or focus group? | Yes | Methods |  |
| What was the duration of the interviews or focus group? | 60 minutes | Methods |  |
| Was data saturation discussed? | No |  |  |
| Were transcripts returned to participants for comment and/or correction? | No |  |  |
| ***Domaine 3: Analysis and Findings*** | | |  |
| ***Data analysis*** | | |  |
| How many data coders coded the data? | 2 | Methods |  |
| Did the authors provide a description of the coding tree? | Open coding is described in the Methods Data Analysis section | Methods |  |
| Were themes identiﬁed in advance or derived from the data? | Derived from the data | Methods |  |
| What software, if applicable, was used to manage the data? | Atlas.ti, Microsoft Word | Methods |  |
| Did participants provide feedback on the ﬁndings? | No |  |  |
| ***Reporting*** | | |  |
| Were participant quotations presented to illustrate the themes/ﬁndings?  Was each quotation identiﬁed? | Quotations provided and identified by occupation | Results |  |
| Was there consistency between the data presented and the ﬁndings? | Yes |  |  |
| Were major themes clearly presented in the ﬁndings? | Yes | Results, Table 2 |  |
| Is there a description of diverse cases or discussion of minor themes? | No |  |  |
