# Supplementary material for: Promoting permanency in families with parental substance misuse: lessons from a process evaluation of a multi-system program
Source: BMC Public Health. 2022 Dec 3;22:2261. doi: 10.1186/s12889-022-14528-4 (PMC9719642; doi:10.1186/s12889-022-14528-4)
Supplement: Supplementary file 2 — Additional file 2: Appendix B. EPIC Process Review: Interview Questions. [file 12889_2022_14528_MOESM2_ESM.docx]

**Appendix B**

**EPIC Process Review: Interview Questions**

**Child Welfare Administration**

| **Readiness** |
| --- |
| What types of workflow practices did you put into place prior to EPIC implementation? |
| **Responsiveness/Adaptability of EPIC Administration** |
| What types of barriers did you observe? How did you address them? |
| What types of strengths (facilitators) did you observe? |
| **Implementation/Collaboration** |
| Walk me through the full process of offering a family the EPIC program. Who completes UNCOPE assessments? How are decisions to offer services made? |
| In what ways do you share resources or best practices with [details omitted for double-anonymized peer review]? |
| **Peer Recovery Support Implementation** |
| What types of cross-training did PRS receive? Effective? Additional need for cross-training? |
| Have you implemented any agency-wide policies to address the needs of PRS? |
| How successful are current recruitment/retention efforts for PRS? |
| Have you implemented a system to match PRS to families? |
| Any unexpected or surprising observations around PRS implementation/engagement? |
| **FTDC/MAT Implementation** |
| What process is in place for referring participants to Drug Court? |
| What are some barriers to engagement in drug court? |
| What process is in place for referring participants to Drug Court? |
| What are some barriers to engagement in MAT? |
| Any unexpected or surprising observations around FTDC/MAT implementation/engagement? |
| **NPP Implementation** |
| What process is in place for referring kinship caregivers to NPP? |
| What are some barriers to engagement in NPP? |
| Any unexpected or surprising observations around NPP implementation/engagement? |
| **Sustainability/Building Partnerships** |
| What are some key agencies EPIC should partner with? |
| What types of support systems are in place for families that complete EPIC? |

**Service Provider Administration**

| **Readiness** |
| --- |
| What types of cross-training did you receive prior to implementation? Effective? Additional cross-training needs? |
| **Implementation** |
| Tell me about the process of offering the family services. What does that conversation look like? |
| What are families' reactions to being offered the services? |
| In what ways do you share resources or best practices between the two counties? |
| What types of monitoring or supervision policies are in place for PRS? |
| **EPIC Materials** |
| What types of cross-training did PRS receive? Effective? Additional need for cross-training? |
| In what ways do you use the following? How useful are they? If not very useful, how can we improve them?   - Opioid Toolkit: [https:](https://u.osu.edu/epic/epic-enhancing-permanency-in-children-and-families/about/services/supporting-children/)  [details omitted for double-anonymized peer review] - Drug Testing and Child Welfare: [https:](https://u.osu.edu/epic/epic-enhancing-permanency-in-children-and-families/about/services/supporting-children/)  [details omitted for double-anonymized peer review] - Family Treatment Drug Court: [https:](https://u.osu.edu/epic/epic-enhancing-permanency-in-children-and-families/about/services/supporting-children/)  [details omitted for double-anonymized peer review] - MAT: [https:](https://u.osu.edu/epic/epic-enhancing-permanency-in-children-and-families/about/services/supporting-children/)  [details omitted for double-anonymized peer review] - PRS: [https:](https://u.osu.edu/epic/epic-enhancing-permanency-in-children-and-families/about/services/supporting-children/)  [details omitted for double-anonymized peer review] - NPP: [https:](https://u.osu.edu/epic/epic-enhancing-permanency-in-children-and-families/about/services/supporting-children/)  [details omitted for double-anonymized peer review] |
| Have opioid-specific modules been implemented? |
| **NPP** |
| What process is in place for referring kinship caregivers to NPP? |
| When did kinship caregiver support (NPP for kinship caregivers) begin? |
| What are the barriers to accepting NPP? |
| What are the barriers to keeping families engaged in NPP? |
| What steps do you take when you are having difficulty keeping a family engaged? |
| How will you know when a family has successfully completed services? What does that look like? |
| What barriers are limiting the effectiveness of the Kinship Caregiver Support intervention on improving communication among KC, child, and BP? |
| What barriers are limiting the effectiveness of the Kinship Caregiver Support intervention on improving relationships and child attachment among KC, child, and BP? |
| What factors are helping promote the effectiveness of the Kinship Caregiver Support intervention on improving communication among KC, child, and BP? |
| What Factors are helping promote the effectiveness of the Kinship Caregiver Support intervention on improving relationships and child attachment among KC, child, and BP? |

**County Caseworkers**

| **Readiness & Resource Sharing** |
| --- |
| What types of workflow practices did you put into place prior to EPIC implementation? |
| What types of cross-training did you receive prior to implementation? Effective? Additional need for cross-training? |
| **Implementation/Collaboration** |
| Walk me through the full process of offering a family the EPIC program. Who completes UNCOPE assessments? How are decisions to offer services made? |
| Tell me about the process of offering the family services. What does that conversation look like? |
| What are families' reactions to being offered the services? |
| What are the barriers to accepting EPIC? |
| What are the barriers to keeping families engaged in EPIC? |
| What steps do you take when you are having difficulty keeping a family engaged? |
| How will you know when a family has successfully completed services? What does that look like? |
| **Peer Recovery Support Implementation** |
| What process do you use to match EPIC participants to PRS? |
| Have you made any unexpected or surprising observations when matching participants to PRS? |
| **FTDC/MAT Implementation** |
| How do you make referrals to drug court? How do you monitor participant progress/what types of reports do you receive from the courts? |
| What barriers have you observed for engagement in drug court? |
| What facilitators have you observed for engagement in drug court? "Facilitators" include strategies that you (or the participant) have used successfully or participant circumstances that support engagement in drug court. |
| How do you make referrals for MAT? How do you monitor participant progress/what types of reports do you receive from providers? |
| Describe barriers have you observed for engagement/retention in MAT? |
| Describe facilitators that have you observed for engagement/retention in MAT? "Facilitators" include strategies that you (or the participant) have used successfully or participant circumstances that support engagement in MAT. |
| Any additional observations around implementing FTDC and MAT for EPIC participants? |
| **NPP Implementation** |
| How do you make referrals for NPP? How do you monitor participant progress/what types of reports do you receive from [details omitted for double-anonymized peer review]? |
| Describe barriers have you observed for engagement in NPP? |
| Describe facilitators have you observed for engagement in NPP? "Facilitators" include strategies that you (or the participant) have used successfully or participant circumstances that support engagement in NPP. |
| Any additional observations around implementing NPP EPIC participants? |
| **Needs Portal** |
| How does the Needs Portal support collaboration with PRS? If not, why not? |
| What has been your experience with technical assistance? What about adding changes/updating the Needs Portal? |
| How do you think the Needs Portal has influenced the timeliness of service provision to clients? |
| How does the Needs Portal support collaboration with BHS? If not, why not? |
| **EPIC Materials** |
| What types of cross-training did PRS receive? Effective? Additional need for cross-training? |
| In what ways do you use the following? How useful are they? If not very useful, how can we improve them?   - Opioid Toolkit: [https:](https://u.osu.edu/epic/epic-enhancing-permanency-in-children-and-families/about/services/supporting-children/)  [details omitted for double-anonymized peer review] - Drug Testing and Child Welfare: [https:](https://u.osu.edu/epic/epic-enhancing-permanency-in-children-and-families/about/services/supporting-children/)  [details omitted for double-anonymized peer review] - Family Treatment Drug Court: [https:](https://u.osu.edu/epic/epic-enhancing-permanency-in-children-and-families/about/services/supporting-children/)  [details omitted for double-anonymized peer review] - MAT: [https:](https://u.osu.edu/epic/epic-enhancing-permanency-in-children-and-families/about/services/supporting-children/)  [details omitted for double-anonymized peer review] - PRS: [https:](https://u.osu.edu/epic/epic-enhancing-permanency-in-children-and-families/about/services/supporting-children/)  [details omitted for double-anonymized peer review] - NPP: [https:](https://u.osu.edu/epic/epic-enhancing-permanency-in-children-and-families/about/services/supporting-children/)  [details omitted for double-anonymized peer review] |
| **Miscellaneous** |
| Describe any service needs you're unable to easily address? |

**Peer Recovery Supporters**

| **Readiness & Resource Sharing** |
| --- |
| What types of cross-training did you receive prior to implementation? Effective? Additional need for cross-training? |
| **Implementation/Collaboration** |
| What is the process for matching you with a START or EPIC client? |
| How do you communicate with or update the ongoing caseworker? |
| How would you describe the job of a peer recovery supporter for EPIC? What about START? Are there any differences? |
| How many individuals/couples are you currently working with? How manageable is this caseload for you? |
| How often do you see them? |
| What have you observed about willingness/ability to participate in drug court? Barriers? Strengths? |
| What have you observed about willingness/ability to receive MAT? Barriers? Strengths? |
| What barriers have you observed to entering SUD treatment generally (doesn't have to be FTDC or MAT)? |
| What has been helpful in the past for encouraging parents to begin SUD treatment generally (doesn't have to be FTDC or MAT)? |
| Have you worked with families who successfully completed START or EPIC?  If so, how do you terminate the relationship with a client? Do you still act as a support system for them even after they are done with the CSW? |
| **Needs Portal** |
| How does the Needs Portal support collaboration with caseworkers? If not, why not? |
| What has been your experience with technical assistance? What about adding changes/updating the Needs Portal? |
